# Is Chronic Pain Temporal Pattern Associated with Middle-Aged and Older Adults’ Perceptions of their Futures?

**DOI:** 10.5334/hpb.34

**Published:** 2021-12-30

**Authors:** GILLIAN FENNELL, ABBY PUI WANG YIP, M. CARRINGTON REID, SUSAN ENGUÍDANOS, ELIZABETH ZELINSKI, CORINNA LÖCKENHOFF

**Affiliations:** University of Southern California, US; Cornell University, US; Weill Cornell Medical College, US; University of Southern California, US; University of Southern California, US; Cornell University, US

**Keywords:** Future time perspective, chronic pain, temporal pattern, duration, interference

## Abstract

A psychological consequence of chronic pain may be an inappropriately limited future time perspective (FTP) for middle-aged and older adults. FTP is defined as one’s perception of time as limited or expansive. Potentially meaningful measures, like pain temporal pattern, are often ignored in the chronic pain literature. The present study uses secondary data to assess the association between pain temporal pattern and FTP, and the moderating effect of pain duration. Among 140 individuals with chronic pain, there was no significant association between pain pattern and FTP. However, both pain-related activity interference and pain duration were associated with FTP where greater interference predicted more limited FTP (*b* = −0.16, *p* = .03) and longer pain duration contributed to more expansive FTP (*b* = 0.001, *p* = .03). The temporal pattern x pain duration interaction terms were non-significant. We discuss implications, limitations, and future directions of these findings.

It is well-established that chronic pain negatively affects physical functioning and increases mood disorder risk (Blair et al. 2003; Goldenberg 2010; Thomas et al. 2004; Wæhrens, Amris, & Fisher 2010). Lesser known is that pain can hinder individuals’ qualitative perceptions of the future by making it difficult to either envision a future at all or by fostering a pessimistic outlook (Agerström, Stening & Axman 2019; Coyle & Atkinson 2018; Hellström & Carlsson 1997).

To our knowledge, future time perspective (FTP), a subjective assessment of future opportunities and the amount of time one has left to live (Carstensen & Lang 1996), has yet to be measured in a chronic pain sample. One’s perception of future time ranges from ‘limited’ to ‘expansive’: a spectrum that demonstrates strong associations with age, health, and psychological wellbeing. Limited FTP is commonly reported among older adults and/or individuals in poor health, and is associated with a tendency to prioritise emotional wellbeing (Coudin & Lima 2011; Charles & Carstensen 2010; Lang & Carstensen 2002). Conversely, younger, healthier cohorts typically report expansive FTP, making them more likely to engage in long-term planning (Lang & Carstensen 2002; Löckenhoff & Carstensen 2004). Expansive perceptions of future time have also been linked to greater treatment adherence (Sansbury et al. 2014), use of efficacious active and emotion-focused coping strategies (e.g. cognitive reappraisal and exercise; Bolotova & Hachaturova, 2013), feelings of hope, and general optimism (Kooij et al. 2018).

Qualitative research has demonstrated that, regardless of age, the presence of chronic pain contributes to more negative outlooks, particularly when pain is severe and unmanageable (Coyle & Atkinson 2018; Kristiansen et al. 2012). On the other hand, the ability to control or reduce pain alleviates anxiety and boosts optimism—a positive correlate of expansive FTP (Hallberg & Carlsson 1998; Kooij et al. 2018).

There are several aspects of chronic pain that have been measured in previous research. These include pain intensity (i.e., magnitude of pain, often categorized into mild, moderate, and severe; Suso-Ribera et al., 2019), interference (i.e. pain-related limitations to daily function; Barry et al. 2017), sensation (e.g., burning, aching, shooting, etc.; Jensen et al. 2013), location (i.e., referencing body areas where pain is experienced; Konstantinou et al. 2013), duration (i.e., the amount of time individuals have experienced pain; Jess et al. 2021), and so on. Despite this range of available measures, pain intensity and interference are the most commonly assessed correlates of poor wellbeing and life satisfaction (e.g., Hawker et al. 2011; Sánchez-Rodríguez et al. 2020; Stålnacke 2011). However, only measuring these two facets of pain fails to address the holistic pain experience, including how afflicted individuals perceive their futures. A small number of studies have used a lesser known pain measure, *temporal pattern* of pain, and found similar associations with poor well-being and life satisfaction (Liu et al. 2014; Mullady et al. 2011; Wu et al. 2010). This measure categorizes experienced pain into three groups: (1) intermittent pain refers to pain that comes and goes, (2) variable pain is ever-present but fluctuates in intensity, and (3) constant pain is stable in both presence and intensity (Jensen et al. 2006).

Among those with any pain, the intermittent pattern has been associated with less disability, fewer doctor visits, and better emotional wellbeing; whereas those experiencing constant pain describe a poorer quality of life (Attal et al. 2011; Mullady et al. 2011). Intermittent pain has also been shown to be preventable via disengagement with triggering activities, corresponding with mitigated consequences of chronic pain (Hallberg & Carlsson 1998). Alternatively, severe, acute, and/or unmanageable chronic pain (more closely related to constant, or variable pain) contributes to an obstruction to future planning (Agerström et al. 2019; Hellström & Carlsson 1997).

Pain duration may be another important factor predicting individuals’ perceptions of future time. However, the directional effect of pain duration is not certain. For example, individuals often experience feelings of loss and denial directly following pain onset (Dildy 1996) and tend to adapt to limitations with time (Khanom et al. 2020; Sanderson et al. 2011). Therefore, longer pain durations may facilitate more expansive perceptions of the future. In contrast, longer pain experience has been related to greater functional impairment (Davison et al. 2014), worsened pain intensity, and increased pain catastrophizing (Peters et al. 2000); all of which could contribute to the adoption of more limited FTPs.

## PRESENT STUDY

The present study examines the association of pain, particularly pain temporal pattern and pain duration, with future time perspective. This exploratory research will help confirm the utility of the pain temporal pattern measure and spearhead research efforts to further assess the relationship between pain and future thinking, which may drive interventions related to treatment adherence and goal-setting. Despite a lack of clear theoretical predictions, prior work suggests that, relative to individuals with variable or constant pain, individuals who experience pain intermittently may have more expansive perceptions of future time because they tend to have better psychological and physical health outcomes. Further, we assess whether pain duration moderates the possible association between pain temporal pattern and FTP. Lastly, due to the paucity of research on pain pattern, we describe the composition of each pain pattern group and examine whether available socio-demographic (e.g., age, sex, race, education) factors and subjective health significantly predict pain patterns in any direction.

## METHOD

### PROCEDURE

Prior to conducting this study, all procedures were approved by the Cornell University Institutional Review board. A convenience sample of 150 participants was recruited in 2017–2018 from Ithaca, New York and New York City. In Ithaca, participants were recruited via existing participant pools from Cornell University’s Healthy Aging Lab, in-person and electronic announcements (to the Self-Management Resource Center database), and flyers in physical therapy offices, pain clinics, the Office for the Aging, and the Lifelong Senior Center. New York City participants were recruited through local senior centers, the Wright Center on Aging, and the Research Career Institute in the Mental Health of Aging. It is assumed that participants who were recruited at physical therapy offices and pain clinics were accessing pain management supports through these providers prior to their involvement in the study; there were no survey questions on this topic. All eligible participants were at least 45 years old and reported experiencing non-cancer pain on most days for at least three consecutive months in the past year. There was no upper age limit. To ensure their ability to provide reliable self-reports, participants also had to score a 4 or higher on the Six-Item Cognitive Screening Test (Callahan et al. 2002). This test is a well-established assessment that has shown to be valid and reliable for screening participants with significant cognitive impairment in a number of studies (Carpenter et al. 2011; Chen et al. 2010; Wilber et al. 2005).

Prospective participants were contacted by phone to screen for the presence of significant cognitive impairment and to evaluate their eligibility for this study. If eligible, individuals were asked to schedule a time for an in-person or phone-based interview. During each interview, a research assistant read the consent form and survey questions (see Replication Package) aloud. Verbal responses were then marked into a Qualtrics form. These are secondary data originally collected to investigate the affective correlates of chronic pain (Yip et al. 2018; Yip 2019). In addition to the variables relevant to the present study, the interview also included items regarding actual, ideal, and avoided affect, predictions about future pain, coping strategies, and self-continuity.

### MEASURES

#### Pain temporal pattern

Pain temporal pattern was assessed by a single item from the Pain Quality Assessment Scale (Jensen et al. 2006). Participants were given definitions of constant, intermittent, and variable pain and asked to select the one that best represented their pain experience.

#### Future Time Perspective

Future Time Perspective was measured using Carstensen and Lang’s (1996) Future Time Perspective Scale (FTPS). The instrument consists of 10 questions (with 7-point Likert scale responses), asking participants to indicate whether a statement about their future is (1) very untrue for them to (7) very true for them. Statements include “many opportunities await me in the future” and “My future is filled with possibilities.” Of the 10 items, three are reverse-coded (e.g., “I have the sense that time is running out.”) Carstensen & Lang (1996) is an unpublished manuscript, but the scale can be accessed on the Stanford Life-Span Development Laboratory webpage (https://lifespan.stanford.edu/download-the-ftp-scale). Our data suggest that the FTPS is sufficiently reliable with a Cronbach’s alpha equal to .91.

We also considered two additional variables to better understand the effect of pain experience. *Pain duration* was assessed with a single open-ended item: “How many months have you experienced this pain?” *Pain Interference* was also assessed with a single item from the SF-12 questionnaire (Ware, Kosinski & Keller 1996). Respondents rated how much their pain interfered with their daily activities on a 5-point Likert scale from “Not at all” to “Extremely” over the past week.

Age, race, sex, educational attainment, subjective health, and average pain intensity were included in Table 2. Respondents self-identified as Black or African American, Asian, Native Hawaiian or other Pacific Islander, Native Indian or Alaskan Native, White, or Other. Education was coded as four levels of educational attainment: high school education or less, a 2-year college degree or some college, a 4-year bachelor’s degree, or a graduate degree. Subjective health was measured by a single item from the SF-12 questionnaire (Ware et al. 1996). Participants answered the prompt “In general, would you say your health is:” with one of five options: Excellent, Very good, Good, Fair, or Poor. *Pain Intensity* was measured with a single Likert item from the Pain Intensity Questionnaire within PROMIS (Cella et al., 2010), on which each respondent indicated their average pain from 1 (no pain) to 7 (intense pain) over the prior seven days.

### ANALYSIS

For detailed code, see the STATA.16 .do file included in the Analysis Package.

To build the main effect model assessing the relationship between pain temporal pattern and FTP, we restricted the possible predictors to the four pain variables (i.e. pain time pattern, intensity, interference, and duration) and age. Age was examined because of its consistent association with FTP in extant literature (e.g., Liao & Carstensen 2018). We implemented a stepwise forward selection and generated Akaike’s Information Criteria (AIC) and Bayesian Information Criteria (BIC) fit statistics after every addition. Lower values for both AIC and BIC indicate better model fit (Akaike 1974; Schwarz Gideon 1978); variables that contributed to lower AIC and BIC outputs—and thus improved model fit—were included in the final model. Pain temporal pattern, pain interference, and pain duration were retained in our model. Pain intensity and age were excluded. See Table 1 for the final model. Subjective health is also a strong predictor of FTP (Kooij & Van De Voorde 2011), but was excluded because pain is a well-known factor in self-rated health reporting (Chireh & D’Arcy 2018); including this variable would have limited our understanding of the relationship between pain and FTP.

Following the initial analysis, we created two binary variables to represent the “variable” and “constant” temporal pattern groups. In two separate analyses, a pain duration × temporal pattern interaction term was included to assess the moderating effect of pain duration on our relationship of interest. The first referred to variable pain and the second to constant pain; participants reporting intermittent pain served as the reference group in both analyses. In line with the main effect model, the two moderation models also controlled for pain interference.

Table 2 describes the composition of each of the pain pattern (intermittent, variable, and constant) subgroups with results from bivariate logistic regressions. These regressions assessed the relationships between the listed demographic, health, and pain-related factors and the variable and constant pain patterns, respectively. The intermittent pain group served as the reference group.

## RESULTS

One-hundred fifty participants were initially recruited, however two were excluded due to serious illness and an additional eight were excluded from the final analytic sample due to missing pain duration data. Of the remaining 140, the majority (76%) were female, 85% self-identified as European American, 7% as African American, 2% as Asian, and 5.7% as “Other.” Participants were between the ages of 45 and 94 (*M*age = 64.5; *SD*age = 12.1). Over a third (38%) had a graduate degree, 35% completed a 4-year college degree, 25% finished a 2-year college degree or completed some college, and 7% received a high school diploma or reported less educational attainment. Sample pain characteristics were as follows: pain intensity (*M* = 4.05, *SD* = 1.27), pain interference (*M* = 3.23, *SD* = 0.97), and pain duration (*M* = 129.09, *SD* = 137.3). Pain duration ranged from 3 to 636 months. Information on pain temporal pattern is in Table 2.

The main linear regression model (see Table 1) was statistically significant overall (*F*(4, 135) = 2.71, *p* = .03). However, that finding was driven by FTP’s association with pain interference and pain duration (*b* = −0.16, *t* = −2.15, *p* = .03; *b* = 0.001, *t* = 2.20, *p* = .03). After controlling for these two variables, there was no significant association between pain temporal pattern and FTP (constant: *b* = −0.18, *t* = −0.88, *p* = .38; variable: *b* = −0.10, *t* = −0.60, *p* = .55). Both of the pain time pattern x duration interaction models yielded non-significant results as well. Compared to participants with intermittent pain, pain duration did not moderate perceptions of future time for participants with either variable (*b* = 0.001, *t* = 0.84, *p* = .40) or constant (*b* = 0.001, *t* = 0.66, *p* = .51) pain.

Table 2 shows that participants with high levels of education and better self-reported health were 51% and 43% less likely to report variable pain relative to intermittent pain (*OR* = 0.49, *95% CI* [.30 – .80]; *OR* = 0.57, *95% CI* [.38 – .85]). These data are similar for constant pain: highly educated and self-perceptively healthy individuals were 55% and 59% less likely to report constant pain than intermittent pain (*OR* = 0.45, *95% CI* [.26 – .79]; *OR* = 0.41, *95% CI* [.23 – .73]). Further, those with high pain interference were 64% more likely to report variable pain, and women were 69% less likely to report constant pain relative to intermittent pain.

## DISCUSSION

Findings revealed that regardless of whether an individual experienced constant, variable, or intermittent pain, their FTP scores were statistically equivalent. What matters more in predicting FTP is the amount of time individuals experience chronic pain and whether that pain hinders daily activity. However, these were independent main effects; interaction terms assessing the moderating effect of pain duration on the relationship between pain temporal pattern and FTP were not significant.

The lack of support for our hypothesis regarding pain temporal pattern suggests that our empirically-informed hypothesis was incorrect. The reasons we cited in the introduction for why we believed intermittent pain would be associated with more expansive FTPs relative to variable and constant pain were based on other pain and health factors. For example, intermittent pain has been associated with less disability and better self-rated health (Attal et al. 2011; Mullady et al. 2011). However, while our data demonstrate a similar association between temporal pattern and subjective health (*r* = −0.29, *p* = .0004), there is no significant relationship between pain pattern and activity interference (*r* = 0.10, *p* = .26). Thus, the link between pain pattern and disability is not as consistent across samples as we originally believed.

The independent effects of both pain duration and pain interference on FTP have support from adaptation and disability literature unrelated to FTP. With respect to our pain duration finding, it is possible that, as Khanom et al. (2020) and Sanderson et al. (2011) suggested, experiencing chronic pain for a longer duration facilitates better psychological adaptation and greater pain acceptance. Psychological adaptation to chronic stressors has also been associated with to more expansive perceptions of the future (Livneh & Martz 2007; Martz 2004). In contrast to literature suggesting that longer experience with chronic pain corresponds with worse pain-related health outcomes (e.g. Davison et al. 2014; Peters et al. 2000), longer pain duration was not associated with increases in pain intensity or interference in our sample. Previous research supports our pain interference findings as well: pain-related disability has been found to truncate both perceptions of future time (Livneh 2013) and subjective life expectancy (a similar construct assessing how long individuals expect to life; Mirowsky & Ross 2000).

Provided that future research replicates our pain interference finding, interventions to expand FTP should be targeted at individuals who experience high levels of pain-related disability. Physician or therapist-led goal-setting, monitoring, and implementation interventions have been shown to be effective for improving self-efficacy and treatment adherence among people with chronic pain (Coppack, Kristensen & Karageorghis 2012; Baird et al. 2021). Goal setting and monitoring allow individuals to actively modulate their FTP by imagining a positive, attainable future and developing an action plan to actualize it. A recent meta-analysis reported that goal monitoring and implementation—not simply goal setting—mediated the relationship between time perspective and positive life outcomes, including improved physical health and psychological wellbeing (Baird et al. 2021). This suggests that physicians and therapists who initiate goal interventions must follow up with their patients to ensure that (a) their health and pain-related goals are realistic and attainable, and (b) they are actively implementing the goals they set.

## LIMITATIONS

Our findings should be considered amidst limitations. Our use of single item measures for pain temporal pattern and pain interference prevented us from reporting reliability statistics. With respect to validity, pain interference demonstrates concurrent validity through its expected associations with pain intensity (*r* = 0.38, p < .0001) and subjective health (*r* = −0.38, p < .0001), however, the validity of our pain temporal pattern measure is less certain. As mentioned previously, our hypothesis was predicated on the association between pain interference and pain temporal pattern, but there was no such association in our data. Thus, our pain pattern measure may not be adequately valid. However, the true relationship between pain pattern and activity interference is still unclear, especially in the context of different pain conditions. For example, in those with knee osteoarthritis, unpredictable intermittent pain seems to be more physically and psychologically distressing than constant pain, whereas constant, unrelenting pain is more problematic for chronic pancreatitis patients (Liu et al. 2014; Mullady et al. 2011). Since our sample was not limited to a specific pain condition, this may explain the lack of association between pain pattern and interference.

### FUTURE DIRECTIONS

Due to the paucity of research assessing the relationship between pain and FTP, basic research is needed to establish a stronger empirical connection between these two variables. For example, future researchers may consider comparing FTP scores among individuals with and without pain, then, in subsequent studies with larger samples, they may assess the influence of different aspects of chronic pain (e.g., intensity and interference) on FTP. If researchers wish to reexamine the relationship between pain temporal pattern and FTP (with two controls), we suggest—based on a conducted power analysis—a minimum sample size of 310 participants to detect an effect of *p* = .05.

Future research should also be attentive to two pain-related variables that we did not account for in our models: pain condition and location. Using what was gained from the present study, these factors may be investigated as moderating variables in the relationship between pain interference and FTP. Different pain conditions and locations have different prognoses and associated disability risks (Barry et al. 2017; Hicks et al. 2008; Hider et al. 2015). For example, relative to arm or shoulder pain, lower extremity (e.g., in legs, hips, and feet) pain may be more disabling because it makes ambulation more taxing, contributing to greater pain interference and possibly more limited FTP. Miró and colleagues (2014b) reported individuals with lower extremity muscular dystrophy (MD) pain had greater self-reported disability compared to MD patients with back or arm pain (Miró et al. 2014b). These differences in disability reporting by pain location may translate to systematic differences in the ways individuals perceive their futures. In a similar study of individuals with chronic pain due to spinal cord injury, Miró et al. (2014a) emphasized the importance of measuring pain intensity in specific pain sites—not simply average pain intensity—when estimating the effect of pain on physical and *psychological* functioning. Pain intensity in the legs and lower back showed the strongest associations with pain interference and mental health (Miró et al. 2014a). Future research should continue to explore differential consequences of these pain-related variables on psychological constructs like FTP.

## CONCLUSION

Contrary to what authors hypothesized, this study suggests that pain temporal pattern does not influence individuals’ FTPs. This null association between pain temporal pattern and FTP may be an accurate representation of a non-significant relationship, or could be due to our use of suboptimal measures in a sample of participants with an array of chronic pain conditions. We offer insight into the relationships between FTP, pain duration, and activity interference, respectively. Our findings regarding pain duration support the empirically-based notion that longer experiences with chronic pain facilitate enhancements in psychological adaptation and wellness, translating to more expansive FTP. Further, the association between pain interference and limited FTP is supported by disability literature. Before clinicians roll out interventions to expand FTP in those with pain-related disability, we encourage future work that focuses on developing stronger empirical ties between pain and FTP. Following basic comparative studies between groups with and without pain, researchers should assess the moderating role of pain location and condition on the relationship between pain interference and FTP. Pain intensity and interference are well-established predictors of physical and psychological outcomes, but when used alone, they may provide an incomplete picture of an individual’s pain experience.

## Figures and Tables

**Table 1:** The main linear regression predicting FTP.

	MAIN EFFECT REGRESSION MODEL		
	B		95% CI
*Variables*			
Pain temporal pattern			
Intermittent pain (ref)			
Variable pain	−0.1		[−0.44, 0.23]
Constant pain	−0.18		[−0.60, 0.23]
Pain duration (months)	0.001	*	[0.0001, 0.002]
Pain interference (out of 5)	−0.16	*	[−0.31, −0.012]
Constant	3.41	***	[2.89, 3.94]
R2	0.074		

* p < .05;

** p < .01;

*** p < .001.

**Table 2:** Pain temporal pattern group compositions and results from bivariate logistic regressions using socio-demographic/health factors to predict pain pattern group status. Intermittent pain is the reference group.

	PAIN PATTERN: INTERMITTENT MEAN (SD)	PAIN PATTERN: VARIABLE M (SD)	ODDS RATIO [95% CI]	PAIN PATTERN: CONSTANT MEAN (SD)	ODDS RATIO [95% CI]
**Group N**	40	73		27	
**Age**	65.7 (11.65)	61.59 (11.22)	0.97 [.94 – 1.00]	67.44 (13.93)	1.01 [.97 – 1.05]
**Sex (% female)**	80%	80.82%	1.05 [.40 – 2.78]	55.56%	0.31 [.11 – .92]**
**Race (% white)**	90%	83.56%	0.56 [.17 – 1.88]	81.48%	0.49 [.12 – 2.02]
**Education (out of 4)**	3.38 (.74)	2.89 (.91)	0.49 [.30 – .82]**	2.66 (1.14)	0.45 [.26 – .79]**
**Subjective Health (out of 5)**	3.33 (1.02)	2.75 (1.00)	0.57 [.38 – .85]**	2.48 (0.89)	0.41 [.23 – .73]**
**Pain Intensity (out of 7)**	3.85 (1.33)	4.05 (1.22)	1.14 [.84 – 1.56]	4.33 (1.30)	1.33 [.91 – 1.95]
**Pain Interference (out of 5)**	2.98 (1.03)	3.38 (0.84)	1.64 [1.05 – 2.54]*	3.19 (1.14)	1.21 [.76 – 1.92]
**Pain Duration (in months)**	95.7 (122.6)	147.6 (140.6)	1.00 [1.00 – 1.01]	128.4 (144.2)	1.00 [1.00 – 1.01]
**Future Time Perspective (out of 7)**	3.05 (.73)	2.94 (.81)	0.84 [.51 – 1.37]	2.87 (1.13)	0.80 [.46 – 1.38]

* p < .05,

** p < .01.
